# Acute Rheumatic Fever and Rheumatic Heart Disease Among Children — American Samoa, 2011–2012

**Published:** 2015-05-29

**Authors:** Amanda Beaudoin, Laura Edison, Camille E. Introcaso, Lucy Goh, James Marrone, Amelita Mejia, Chris Van Beneden

**Affiliations:** 1 Epidemic Intelligence Service, CDC; 2 Pennsylvania Center for Dermatology, Philadelphia; 3 SWLA Center for Health Services, Lake Charles, Louisiana; 4 Lyndon B. Johnson Tropical Medical Center, Department of Pediatrics, American Samoa; 5 Division of Bacterial Diseases, National Center for Immunization and Respiratory Diseases, CDC

Acute rheumatic fever is a nonsuppurative, immune-mediated consequence of group A streptococcal pharyngitis (strep throat). Recurrent or severe acute rheumatic fever can cause permanent cardiac valve damage and rheumatic heart disease, which increases the risk for cardiac conditions (e.g., infective endocarditis, stroke, and congestive heart failure) (1,2). Antibiotics can prevent acute rheumatic fever if administered no more than 9 days after symptom onset. Long-term benzathine penicillin G (BPG) injections are effective in preventing recurrent acute rheumatic fever attacks and are recommended to be administered every 3–4 weeks for 10 years or until age 21 years to children who receive a diagnosis of acute rheumatic fever (3). During August 2013, in response to anecdotal reports of increasing rates of acute rheumatic fever and rheumatic heart disease, CDC collaborated with the American Samoa Department of Health and the Lyndon B. Johnson Tropical Medical Center (the only hospital in American Samoa) to quantify the number of cases of pediatric acute rheumatic fever and rheumatic heart disease in American Samoa and to assess the potential roles of missed pharyngitis diagnosis, lack of timely prophylaxis prescription, and compliance with prescribed BPG prophylaxis. Using data from medical records, acute rheumatic fever incidence was calculated as 1.1 and 1.5 cases per 1,000 children aged ≤18 years in 2011 and 2012, respectively; 49% of those with acute rheumatic fever subsequently received a diagnosis of rheumatic heart disease. Noncompliance with recommended prophylaxis with BPG after physician-diagnosed acute rheumatic fever was noted for 22 (34%) of 65 patients. Rheumatic heart disease point prevalence was 3.2 cases per 1,000 children in August 2013. Establishment of a coordinated acute rheumatic fever and rheumatic heart disease control program in American Samoa, likely would improve diagnosis, treatment, and patient compliance with BPG prophylaxis.

Acute rheumatic fever is no longer a nationally notifiable disease in the United States, and its annual incidence in the continental United States declined in the late 20th century to approximately 0.04–0.06 cases per 1,000 children (4). Exceptions to these low acute rheumatic fever incidence rates in the United States include Samoan persons living in Hawaii and residents of American Samoa, an American territory in the South Pacific (5,6). Acute rheumatic fever rates in Hawaii have been as high as nearly 0.1 cases per 1,000 children, with even higher rates among persons of Samoan and Hawaiian ethnicity (5). Acute rheumatic fever occurs most commonly among children aged 5–15 years.

Pediatric cases of acute rheumatic fever and rheumatic heart disease were defined as physician-diagnosed acute rheumatic fever or rheumatic heart disease among patients aged ≤18 years who had sought care during 2011–2012 at the hospital in American Samoa. *International Classification of Diseases, Ninth Revision* (ICD-9) codes and BPG prophylaxis registries including patients currently receiving BPG treatment at the hospital were used to identify cases of acute rheumatic fever and rheumatic heart disease during 2011–2012 and to estimate the August 2013 point prevalence of rheumatic heart disease. Acute rheumatic fever diagnostic criteria included classic “Jones criteria” until summer 2012 (7), after which more sensitive Australian and New Zealand guidelines for high-risk areas were used (8). Case finding for inpatients with diagnoses during 2011–2012 was conducted by using ICD-9 codes (390–398). In addition, hospital patient registries for BPG prophylaxis were reviewed to identify additional acute rheumatic fever and rheumatic heart disease patients. Duplicate cases were excluded. Medical records for all identified patients were reviewed to verify acute rheumatic fever or rheumatic heart disease diagnoses and BPG prophylaxis noncompliance, which included recorded missed or late doses. Case-finding using hospital BPG prophylaxis registries was conducted to determine the number of children known to be living with rheumatic heart disease at the time of the study. Acute rheumatic fever incidence (2011–2012) and rheumatic heart disease point prevalence (August 2013) were calculated by using 2010 U.S. Census Bureau data (American Samoa pop. = 55,519, including 24,652 persons aged ≤18 years).

Acute rheumatic fever incidence was 1.1 and 1.5 cases per 1,000 children, for 2011 and 2012, respectively. Of 65 children with physician-diagnosed acute rheumatic fever during 2011–2012, a total of 32 (49%) subsequently received a diagnosis of rheumatic heart disease. Acute rheumatic fever patients were predominantly male (60%); median age at acute rheumatic fever diagnosis was 11 years (range: 2–18 years) (Figure). The 41 patients with available data were of Polynesian (98%) or Fijian (2%) origin. Twelve (18%) patients had a diagnosis of pharyngitis noted in the medical record during the 6 weeks preceding acute rheumatic fever or rheumatic heart disease diagnosis. Noncompliance with post–acute rheumatic fever prophylaxis with BPG was noted for 22 (34%) patients.

Among 32 rheumatic heart disease patients with data, 21 (66%) received a diagnosis of rheumatic heart disease without a previous acute rheumatic fever diagnosis noted in the medical record, indicating that certain patients did not seek care or did not receive a diagnosis until after the disease had progressed. The point prevalence of rheumatic heart disease was 3.2 cases per 1,000 children in August 2013. Of 34 pharyngitis diagnoses made during 2011–2012 and reviewed in acute rheumatic fever patient records, three (9%) were made using rapid antigen detection testing, 15 (44%) were made using throat culture, and 16 (47%) were made without any diagnostic testing.

## Discussion

In addition to causing pharyngitis, pyoderma, and severe invasive disease (e.g., streptococcal toxic shock syndrome and necrotizing fasciitis), group A streptococcal organisms can trigger postinfection syndromes that result from a crossreaction between patient antibodies to bacterial surface proteins and cardiac, neuronal, and synovial tissues (9). Acute rheumatic fever, characterized primarily by carditis, chorea, and polyarthritis, occurs a minimum of 2–3 weeks after an episode of untreated or inadequately treated pharyngitis. Acute rheumatic fever does not cause lasting damage to the nervous tissue or joints. However, damage to heart valves can be irreversible and is worsened by repeat episodes of acute rheumatic fever (1,3). Permanent valvular damage, or rheumatic heart disease, increases the risk for infective endocarditis, stroke, heart failure, and premature death, and might necessitate valve replacement surgery (2). Because pharyngitis and acute rheumatic fever are most common in children, the recurrence of acute rheumatic fever, and, thus, the risk for developing rheumatic heart disease, can continue into adolescence and young adulthood.

This investigation highlights a long-standing disparity in the acute rheumatic fever and rheumatic heart disease rates between children in American Samoa and children in the continental United States. In August 2013, rheumatic heart disease point prevalence in American Samoa (3.2 per 1,000 children) was approximately 10 times that previously estimated for industrialized countries (0.3 per 1,000 children) (2). With improved diagnosis and treatment of group A streptococcal pharyngitis, the United States and other industrialized countries have seen a steep decline in rheumatic heart disease prevalence since the mid-20th century. However, in some parts of the world, rheumatic heart disease is the most common cardiac disease of children and young adults (3). The highest rheumatic heart disease rates occur in sub-Saharan Africa, with an estimated 5.7 cases per 1,000 children aged 5–14 years, and in the Pacific region and indigenous populations of Australia and New Zealand, with 3.5 cases per 1,000 (2).

Multiple factors influence rates of acute rheumatic fever and rheumatic heart disease, including host immune factors and lifestyle (e.g., crowding or access to health care), as well as the biologic characteristics of circulating group A streptococcal strains (1). However, opportunities for prevention exist and include improving access to medical care and using evidence-based strategies to identify and treat group A streptococcal pharyngitis early (primary prevention) and diagnose and prevent recurrent acute rheumatic fever and rheumatic heart disease (secondary prevention) (3).

The World Health Organization recommends community-based acute rheumatic fever and rheumatic heart disease control programs, which include penicillin prophylaxis after an acute rheumatic fever diagnosis to prevent recurrent acute rheumatic fever and rheumatic heart disease (1). Coordinated control programs increase acute rheumatic fever and rheumatic heart disease awareness among patients and the community, improve coverage and compliance with penicillin prophylaxis and medical care, and decrease the rate of recurrent disease (3). Current programs are diverse in their delivery and complexity and include patient registries maintained by health care personnel, community-based prophylaxis, monitoring of medical needs (e.g., echocardiography appointments) and prophylaxis compliance, and education about the importance of prompt diagnosis of group A streptococcal pharyngitis (3). Programs in other countries have been shown to reduce morbidity, disability, and mortality from acute rheumatic fever and rheumatic heart disease (1). Before the decline in acute rheumatic fever incidence in the United States, certain states had prioritized streptococcal disease control and managed control programs.

The morbidity typically associated with rheumatic heart disease, and the disparity between rates in American Samoa and the continental United States, warrant discussion of coordinated control and mandatory public health reporting of acute rheumatic fever and rheumatic heart disease cases in American Samoa. A rheumatic heart disease control program ideally would be operated with local staff members and include measures demonstrated to be successful in controlling acute rheumatic fever and rheumatic heart disease in other high-risk areas, with particular emphasis on timely diagnosis and treatment of group A streptococcal pharyngitis (3). In American Samoa, families often choose traditional remedies over medical care, and this study found that few patients with acute rheumatic fever had a recent diagnosis of pharyngitis. In addition, hospital physicians often rely on clinical, rather than laboratory, diagnosis of pharyngitis. Although penicillin prophylaxis is the only proven cost-effective secondary rheumatic heart disease prevention method, education of health care providers about adherence to clinical practice guidelines for pharyngitis diagnosis and treatment is crucial for acute rheumatic fever and rheumatic heart disease prevention (10).

The findings in this report are subject to at least three limitations. First, this study is likely affected by ascertainment bias, because it only reports acute rheumatic fever patients who sought care at the hospital. Those using traditional remedies for acute rheumatic fever symptoms and patients with mild disease might not seek care. Second, despite multiple case-finding modalities (i.e., registries and medical billing), physicians at the hospital do not assign ICD-9 codes and certain acute rheumatic fever diagnoses might have been missed by the coding staff. The pediatric BPG registry included only currently treated patients. Patients treated during 2011–2012 might have been removed from the registry because of death or emigration. In addition, if not in the adult registry, patients who transitioned from the pediatric to adult medicine service might have been lost to follow-up, and although the hospital serves the majority of residents, a limited number of persons might go off-island for health care. Therefore, this report likely underestimates the number of cases of pediatric acute rheumatic fever and rheumatic heart disease in American Samoa. Finally, medical records were not reviewed for concordance with acute rheumatic fever and rheumatic heart disease diagnostic criteria, potentially affecting the sensitivity and specificity of case ascertainment.

What is already known on this topic?

Inadequately treated group A streptococcal pharyngitis can lead to development of acute rheumatic fever and subsequent rheumatic heart disease, both of which are found at high rates among children living in the South Pacific. Long-term penicillin injections are effective in preventing recurrent acute rheumatic fever attacks and subsequent development of rheumatic heart disease.

What is added by this report?

This report describes a continued high incidence of acute rheumatic fever and prevalence of rheumatic heart disease in American Samoa. In August 2013, rheumatic heart disease point prevalence (3.2 per 1,000 children) was approximately 10 times that estimated for industrialized countries. The report also highlights the extent to which missed diagnoses, missed opportunities for treatment, and treatment noncompliance might contribute to the high rate of rheumatic heart disease.

What are the implications for public health practice?

Efforts to improve pharyngitis diagnosis and treatment and compliance with penicillin prophylaxis might reduce the burden of acute rheumatic fever and rheumatic heart disease among children in American Samoa. These goals might be effectively met by establishment of a coordinated disease control program.

Rheumatic heart disease is expected to cause considerable lifelong morbidity in American Samoa, where it is approximately 10 times more common than in the continental United States. Recommendations to curb rheumatic heart disease in American Samoa are manifold, including improving pharyngitis diagnosis and treatment with concurrent efforts to improve patient compliance with BPG prophylaxis. These goals might be met efficiently and cost-effectively by establishment of a coordinated acute rheumatic fever and rheumatic heart disease control program.

## Figures and Tables

**FIGURE:**
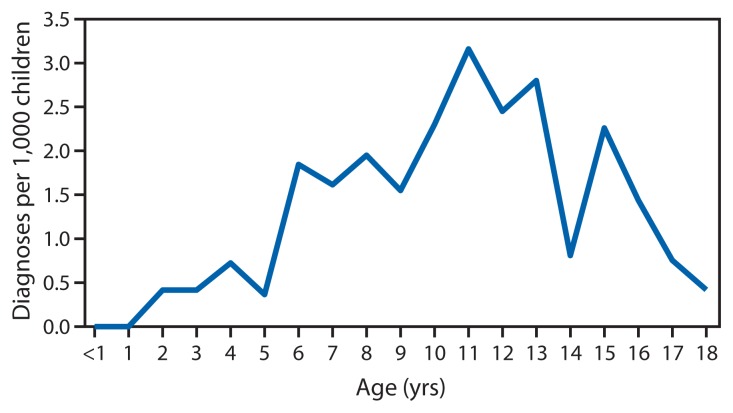
Average annual rate of acute rheumatic fever diagnoses per 1,000 children, by age — American Samoa, 2011–2012
